# Hepatitis B Vaccine Refusal Trends in Washington, DC, Newborns, 2017-2022

**DOI:** 10.1001/jamanetworkopen.2024.21202

**Published:** 2024-07-11

**Authors:** Y. Tony Yang, Timothy F. Leslie, Paul L. Delamater

**Affiliations:** 1 Center for Health Policy and Media Engagement, The George Washington University, Washington, DC; 2 Cancer Center, The George Washington University, Washington, DC; 3 Department of Geography and Geoinformation Science, George Mason University, Fairfax, Virginia; 4 Department of Geography, University of North Carolina at Chapel Hill, Chapel Hill

## Abstract

This cross-sectional study evaluates trends in rates of hepatitis B vaccine birth dose refusals in Washington, DC, from 2017 to 2022.

## Introduction

Hepatitis B affects about 1000 US infants through mother-to-child transmission annually, necessitating immediate birth vaccination to provide 70% to 95% protection and prevent severe disease.^1^ Historically, newborn hepatitis B vaccination rates have been lower than desired, with coverage of the birth dose below 77% among children born during 2017 and 2018.^2^ In response, the Advisory Committee on Immunization Practices (ACIP) revised the vaccination guidelines in January 2018, recommending that medically stable newborns weighing at least 2000 g and born to hepatitis B surface antigen (HBsAg)–negative mothers should receive their first dose within 24 hours of birth.^3^ The new guidelines were more explicit about administering the birth dose within 24 hours and removed the previously permissive language allowing for delay.^4^ The 2018 guideline changes were followed by the COVID-19 pandemic in 2020, causing reduced uptake of routine childhood vaccines.^5^ It is uncertain whether the pandemic affected the rate of hospital-administered birth doses of the hepatitis B vaccination. Our study analyzed trends in hepatitis B birth dose refusals in Washington, DC, hospitals from 2017 to 2022, after the revised guidelines and during the pandemic.

## Methods

This retrospective repeated cross-sectional study analyzed electronic records for newborn hepatitis B vaccinations from the DC Department of Health Vital Statistics Division from January 1, 2017, to December 31, 2022. The analysis examined the percentage of hepatitis B vaccine refusals for the entire cohort and by the mother’s self-reported race (Black, White, and other [Alaska Native or American Indian, Asian, Native Hawaiian or Other Pacific Islander, multiracial, and unknown or not reported]). Analysis was restricted to births at 6 local hospitals with documented hepatitis B vaccine refusal status and a valid birth date. The study was deemed exempt by the George Washington University institutional review board because deidentifed data were used. The study followed the STROBE reporting guideline. R, version 4.3.2 was used for analysis.

## Results

Of the 76 194 recorded births, we excluded 757 nonhospital births, 639 with missing refusal information, and 135 lacking a valid date, leaving 74 660 for analysis (40.8% of mothers were Black; 41.4%, White; and 17.9%, other race). The overall hepatitis B refusal rate decreased from 12.1% of 10 982 births in 2017 to 3.5% of 11 304 births in 2022, with the most substantial declines occurring in 2019 and 2020 (Table). Weekly refusal rates showed a gradual, steady decline starting in early 2018, with a more significant decrease in late 2019 and early 2020 before stabilizing in mid-2020 (Figure). White mothers had the highest initial refusal rates but had the earliest declines. Black mothers and those with other race had similar trends in refusals over time, with those with other race consistently having lower refusal rates than the other groups.

**Table.** Annual Number of Births and Rates of Mother’s Refusal of Hepatitis B Vaccination at Birth in Washington, DC, 2017-2022

Births	Vaccine refusals, No./total No. of births (%)					
	2017	2018	2019	2020	2021	2022
Total	1329/10 982 (12.1)	1627/14 527 (11.2)	1116/13 286 (8.4)	504/12 289 (4.1)	454/12 272 (3.7)	396/11 304 (3.5)
By mother’s race						
Black	496/4409 (11.2)	719/6234 (11.6)	579/5504 (10.5)	235/5012 (4.7)	178/4822 (3.7)	167/4449 (3.7)
White	630/4273 (14.7)	666/5537 (12.1)	366/5442 (6.7)	220/5260 (4.2)	240/5453 (4.4)	175/4933 (3.5)
Other^a^	203/2300 (8.8)	242/2756 (8.8)	171/2340 (7.3)	49/2017 (2.5)	36/1997 (1.8)	54/1922 (2.8)
Exclusions	1016	90	113	117	89	109

^a^ Other race included but was not limited to Alaska Native or American Indian, Asian, Native Hawaiian or Other Pacific Islander, multiracial, and unknown or not reported.

**Figure.**
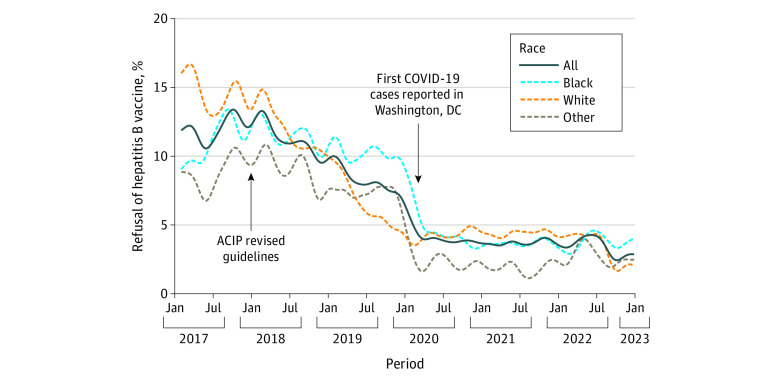
Refusal of Hepatitis Vaccination at Birth in Washington, DC, 2017-2022 Weekly data are shown with a 3-month smoothing window. Other race included but was not limited to Alaska Native or American Indian, Asian, Native Hawaiian or Other Pacific Islander, multiracial, and unknown or not reported.

## Discussion

The substantial decline in newborn hepatitis B vaccine refusals in Washington, DC, aligns with the Healthy People 2030 target of 90% coverage.^6^ This reduction suggests that the updated ACIP guidelines likely played a role in this achievement, with equitable benefits across racial groups.

Despite the pandemic disrupting routine childhood vaccinations,^5^ hepatitis B vaccination efforts remained strong, with sustained low refusal rates in 2021 and 2022, especially for hospital-based birth doses. This contrasts with the broader decline in routine vaccinations, suggesting that the inpatient setting may have contributed to maintaining high hepatitis B birth dose coverage. While vaccine access issues affected routine vaccinations in 2020, access improved in 2021; however, challenges persisted, likely due to increased vaccine hesitancy. Further research is needed to investigate the role of the hospital setting in sustaining hepatitis B vaccination rates during the pandemic.

Limitations include missing data on maternal HBsAg status, infant birth weight, and medical stability and a focus on the Washington, DC, area, which may limit generalizability. As the analysis started a year before the ACIP changes, whether the decline in refusals was due to the new guidelines, a pre-existing trend, or hospital-wide quality improvements is unclear.

## Notes

Supplement. Data Sharing Statement
