# Supplementary material for: Hepatitis B Vaccine Refusal Trends in Washington, DC, Newborns, 2017-2022
Source: JAMA Netw Open. 2024 Jul 11;7(7):e2421202. doi: 10.1001/jamanetworkopen.2024.21202 (PMC11240185; doi:10.1001/jamanetworkopen.2024.21202)
Supplement: Supplement. — Data Sharing Statement [file jamanetwopen-e2421202-s001.pdf]

Data Sharing Statement

Yang. Hepatitis B Vaccine Refusal Trends in Washington, DC, Newborns, 2017-2022. *JAMA Netw Open*. Published July 11, 2024. doi:10.1001/jamanetworkopen.2024.21202

Data
Data available: Yes
Data types: Deidentified participant data
How to access data: ytyang@gwu.edu
When available: With publication

Supporting Documents
Document types: None

Additional Information
Who can access the data: Y. Tony Yang
Types of analyses: for research purpose
Mechanisms of data availability: with a signed data access agreement
